# Mechanical and Thermal Characterization of Annealed Oriented PAN Nanofibers

**DOI:** 10.3390/polym15153287

**Published:** 2023-08-03

**Authors:** Jaymin Vrajlal Sanchaniya, Inga Lasenko, Sai Pavan Kanukuntala, Hilary Smogor, Arta Viluma-Gudmona, Andrejs Krasnikovs, Igors Tipans, Valters Gobins

**Affiliations:** 1 Mechanics and Biotextile Research Laboratory, Riga Technical University, 3/3-20 Pulka Street, LV-1007 Riga, Latvia; inga.lasenko@rtu.lv (I.L.);; 2 Department of Theoretical Mechanics and Strength of Materials, Riga Technical University, 6B Kipsala Street, LV-1048 Riga, Latvia; 3 NETZSCH Instrumenty, Halicka 9, 31-036 Krakow, Poland; 4 Laboratory of Environmental Genetics, Institute of Biology, Faculty of Biology, Latvian University, Jelgavas Street 1, LV-1004 Riga, Latvia

**Keywords:** electrospinning, annealing, oriented nanofibers, mechanical, thermal, thermogravimetric analysis, differential scanning calorimetry

## Abstract

Polyacrylonitrile (PAN) nanofibers have extensive applications as filters in various fields, including air and water filtration, biofluid purification, and the removal of toxic compounds and hazardous pollutants from contaminated water. This research focuses on investigating the impacts of annealing on the mechanical and thermal characteristics of oriented PAN nanofibers produced through the electrospinning of a PAN solution. The nanofiber mats were subjected to annealing temperatures ranging from 70 °C to 350 °C and characterized using a tensile test machine, thermogravimetry, differential scanning calorimetry, and scanning electron microscopy (SEM). The study aimed to examine the tensile strength in the transverse and longitudinal directions, Young’s modulus, and glass transition temperatures of PAN nanofiber mats. The results indicate that, upon annealing, the diameter of the nanofibers decreased by approximately 20%, while the tensile strength increased in the longitudinal and transverse directions by 32% and 23%, respectively. Furthermore, the annealing temperature influenced the glass transition temperature of the nanofiber mats, which exhibited a 6% decrease at 280 °C, while the degradation temperature showed a slight increase of 3.5% at 280 °C. The findings contribute to a better understanding of the effects of annealing on PAN nanofiber mats, facilitating their potential for various filtration applications.

## 1. Introduction

Electrospinning is a low-cost method for producing polymer fibers with diameters ranging from tens to a few hundred nanometers. Electrospun nanofibers are used as optical materials [1], sensor materials [2], nanocomposites [3,4], tissue scaffolds [5], wound treatment [6], drug delivery technologies [7,8,9], filtration [10,11,12,13], protective gear [14], and smart textiles [15,16].

Polyacrylonitrile (PAN) is a semicrystalline synthetic polymer that is hydrophobic and insoluble in many solvents, except for certain polar aprotic solvents and mineral salts [17,18]. Although it is a thermoplastic, PAN is essential because it tends to break down before melting at heating rates of <80 °C/min, since the deterioration begins at 250 °C and the degradation temperature is 317 °C [19,20]. Electrospun PAN nanofibers are often used as the foundation for continuous carbon nanofibers through stabilization and carbonation [21], filtration [22], and catalysis applications [23,24].

Various filtering applications use PAN nanofibers, including air and water filtration, biofluid purification, and the removal of toxic substances and hazardous contaminants from polluted water [25,26]. Due to their large surface area and electrostatic capabilities, PAN nanofibers can efficiently catch airborne particles, such as dust and germs, via air filtration, and during water filtration, they can remove pollutants, including toxic substances, chemical compounds, and bacteria [27,28]. PAN nanofibers are biocompatible and ecologically safe, making them suitable for medical and biological applications. For example, they may be used as filter materials in dialysis machines, wound dressings, or scaffolds for tissue engineering [29]. PAN nanofibers offer many benefits over conventional filtration media, making them viable for various filtering applications.

As a result of the random orientation of the fibers and the wide variety of fiber diameters, electrospun fiber mats require enhanced mechanical characteristics, which limits their practical applications. As the strength of nanofibers is often insufficient [4,30,31], rotating drum collectors help produce oriented nanofibers, which improve their strength. Therefore, improving the mechanical characteristics of electrospun fiber mats is crucial from an industrial standpoint. In order to use oriented pan nanofibers [4,31], it is important to identify the mechanical properties of the oriented nanofibers in the longitudinal and transverse directions; after testing, the effect of annealing is observed in the transverse direction of the nanofiber mat with oriented nanofibers.

The strength of electrospun nanofibers mainly depends on the polymer type, crystallization rate, and crystallinity level. The strength and deformation mechanisms of nonwoven materials depend on the fibers and bonds (connections or joints of the fibers in a nonwoven structure) [32]. Due to their random fiber alignment, lack of adhesion between fibers, and excessive porosity, the mechanical characteristics of electrospun nanofiber mats often require improvement, thus preventing their potential applications from being expanded [33]. Several techniques have been claimed to enhance the mechanical characteristics of nanofiber membranes produced by electrospinning, including cross-linking, annealing [34], hot stretching [35], hot pressing [36], solvent welding [37], and drawing [38]. Typically, all procedures involve the addition of nanoparticles, which primarily serve to increase strength and are also used to perform mat applications [39]. Nanosized materials, such as carbon nanotubes [40,41] and graphite oxide nanoplatelets [42,43,44], are used to reinforce the strength of a single nanofiber, thus strengthening the membrane, just as short fibers are added to strengthen the structures [45,46]. The addition of nanoparticles while fabricating electrospun nanofibers increases the mechanical properties of polymeric nanofiber mats by 35–65% [40,41,47].

Several groups of researchers have found an increase in the tensile strength of nanofibers upon annealing. Young’s modulus increased from 4.7 GPa to 11.1 GPa due to the annealing of electrospun poly(L-lactic acid) (PLLA) at 75 °C for 24 h [48]. The annealing of CG (chitosan–gelatin) membranes in a vacuum oven at varying temperatures increased Young’s modulus and tensile strength by 1.3 and 1.1 times, respectively [34]. Another study [49] used various temperatures to thermally treat a polymer solution of poly(L-lactic acid) doped with multiwalled carbon nanotubes. The selected temperatures were close to Tg and Tm, and the findings indicated that the annealing temperature must be close to the glass transition temperature. The annealing of PVA membranes at 85 °C and 140 °C increased the yield stress by 54%, while the average Young’s modulus increased by 24.5%, respectively [50].

In conclusion, this research article aims to contribute to the existing body of knowledge on oriented PAN electrospun nanofibers by investigating the impact of annealing at different temperatures. While previous studies have touched upon this subject, limited attention has been given to the specific effects of annealing on oriented PAN nanofiber mats produced through electrospinning and collected on a rotating drum collector. By conducting a comprehensive analysis of the morphology, mechanical characteristics, and thermal properties, this study aims to fill this research gap and provide valuable insights into the behavior of PAN nanofibers under different annealing conditions.

## 2. Materials and Methods

### 2.1. Materials

To study the effects of annealing on oriented polyacrylonitrile (PAN), electrospun nanofibers were produced using PAN powder and N,N-dimethylformamide. Polyacrylonitrile (average MW: 150,000 (typical); CAS number: 25014-41-9) and N,N-dimethylformamide (DMF; ACS reagent (solvent); ≥99.8%; CAS number: 68-12-2) were obtained from Sigma-Aldrich chemicals (Merck KGaA, Darmstadt (64287), Germany).

### 2.2. Fabrication of Oriented PAN Nanofibers

**Figure 1:**
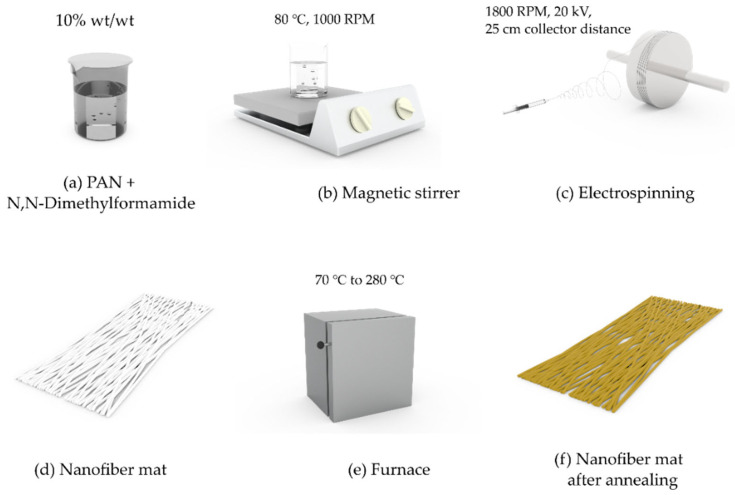
The fabrication process of nanofibers and annealing: (**a**) preparation of the PAN and N,N-dimethylformamide mixture; (**b**) magnetic stirring of the mixture at 80 °C and 1000 rpm; (**c**) fabrication of an electrospun nanofiber mat; (**d**) nanofiber mats; (**e**) annealing nanofiber mats inside the furnace; (**f**) annealed nanofiber mats.

Electrospinning was performed as previously described in [31]. The PAN solution was prepared by adding 10% PAN powder to the solvent (DMF) and mixing it at 80 °C for 10 h with a magnetic stirrer (Thermo Scientific™ Cimarec + TM Stirring Hotplates Series, Waltham, MA, USA) at a stirring speed of 1000 rpm (Figure 1a,b) (ISO 139:2005 [51], where room temperature is 22 ± 1 °C). The solution was kept at room temperature for one hour to eliminate or stabilize air bubbles.

The electrospinning of PAN nanofibers was performed using an electrospinning setup (Fisherbrand TM Single Syringe Pump, a needle-based electrospinning machine, Danbury, CT, USA) with a rotating drum (Shenzhen Tongli Tech Co. Ltd. (D-608), Shenzhen, China; Rotating Collector RC-5000, D140, L50) (Figure 1c) at a room temperature of 22 ± 1 °C. A 10 mL Luer lock syringe and needle (18 Ga with outer diameter of 1.27 ± 0.01 mm and inner diameter of 0.838 ± 0.01 mm) were used. The electrospinning parameters were as follows: a voltage of 20 kV, a flow rate of 1 mL/h, and a distance between the center of the syringe and the rotating collector drum of 25 cm. The rotating speed of the drum collector was constant at 1800 rpm, and aluminum foil (a width of 10 cm and a coating thickness of 35 µm; Vireo.de, Merseburg, Germany) was used on the drum to collect the nanofibers.

For annealing PAN nanofibers (Figure 1d), a Nabertherm LH 30/13 furnace (Nabertherm GmbH, Bahnhofstraße 20 Versand/Wareneingang, Dr. Sasse-Straße 31, Lilienthal, Germany) was used. Each nanofiber mat was placed in the furnace (Figure 1e,f) at room temperature; heated to 70 °C, 140 °C, 210 °C, and 280 °C at a rate of 10 °C/min; held at a constant temperature for 5 min; and then cooled at a rate of 10 °C/min until it reached room temperature naturally. All samples were kept at 22 ± 1 °C and less than 60% relative humidity for 48 h after annealing. Figure 1 summarizes the entire method of spinning nanofibers with annealing used in this work.

To compare the morphology and tensile strength in the longitudinal direction (in the direction of nanofiber orientation), a single electrospun nanofiber mat (440 mm × 50 mm) was cut into six equal parts and tested. An identical nanofiber mat was produced to test the strength of nanofiber mats in the transverse direction (perpendicular to the orientation of the nanofibers).

### 2.3. Morphology

To capture SEM photographs, a Hitachi TM300 tabletop SEM with a magnification of 1500, a vacuum of 10^−2^ Torr, an ion coating with 6 mA, a gold (Au) cover, and a coating thickness of 150 Å was used [31]. Fiber orientation was acquired using the OrientationJ plug-in for the ImageJ program [52,53,54] (ImageJ, National Institutes of Health, Bethesda, MD, USA). The contrast of the SEM images was enhanced to observe the results. The mean diameter and standard deviation of the nanofiber were determined by measuring the diameter of 100 nanofibers randomly selected from three SEM images.

### 2.4. Tensile Test

A Mecmesin Multi-Test 2.5-i tensile testing machine and a 25-N sensor (PPT Group UK Ltd., t/a Mecmesin, Newton House, Spring Copse Business Park, Slinfold, UK) were used to measure the tensile properties. The samples were stored at room temperature according to ISO 139:1973 “Textiles—Standard Environments for Conditioning and Testing”, which specifies a temperature of 21 ± 1 °C, a relative air humidity of 60%, and an atmospheric pressure of 760 mm Hg. The sample size was 50 mm × 10 mm (length × width) according to ASTM D882-18. Five measurements were made to determine the tensile properties. The thickness of the nanofiber mats was measured by a digital micrometer (range: 0–25 mm; Digimatic micrometer, MDC-25PX, code No. 293-240-30, serial No. 71912410, Mitutoyo, Japan). The specimen was cut parallel and perpendicular to the direction of the nanofibers for testing in the longitudinal and transverse directions, respectively. A 50 mm × 40 mm paper template was cut with an inside cut of 30 mm × 20 mm. Using double-sided thin scotch tape (3M Scotch Magic Tape (Matte Finish) 3/4″ × 36 yard Desk Dispenser Refills), both ends of the specimen adhered to the paper template. After attaching the paper and sample to the tensile testing equipment, the sides of the paper template were cut with scissors, as mentioned in [31].

### 2.5. Thermal Test

Thermogravimetric analysis (TGA) was conducted using a TG 209 F1 Libra^®^ thermomicrobalance (NETZSCH, Selb, Germany). Samples weighing 5–6 mg were heated in crucibles made of Al_2_O_3_, and the temperature was increased from 20 °C to 800 °C at a heating rate of 10 °C/min. The samples were exposed to an inert nitrogen atmosphere with a flow rate of 30 mL/min.

**Figure 2:**
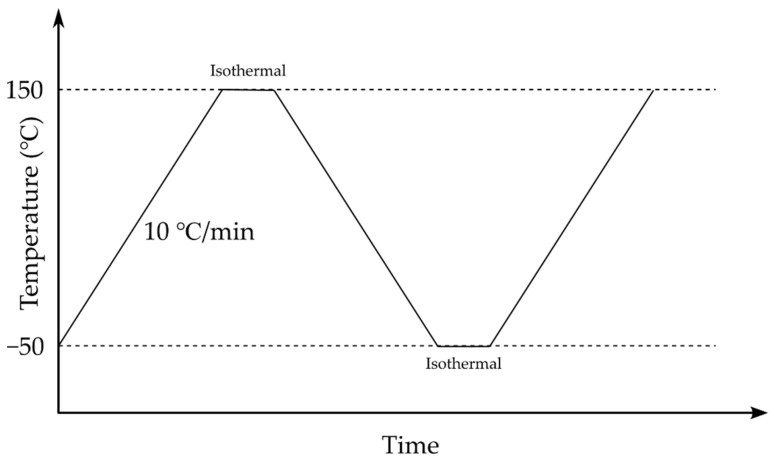
Heating cycle in DSC.

Differential scanning calorimetry (DSC) investigations were carried out using a DSC 214 Polyma differential scanning calorimeter (NETZSCH, Selb, Germany) under a nitrogen (N_2_, CID 947) atmosphere with a flow rate of 30 mL/min. The PAN powder, the PAN nanofiber mat, and the annealed nanofiber mat were precisely cut and placed on the crucible for DSC. Figure 2 represents the heating cycle in the DSC; the first cycle was heated from −50 °C to 150 °C to remove impurities and moisture from the samples, then cooled to −50 °C, and heated again to 150 °C to observe the glass transition temperature. Between each heating and cooling cycle, a 1 min isothermal step was maintained.

DSC was conducted according to ASTM E1356. The samples were heated at a rate of 10 °C/min from −50 °C to 150 °C, as previously described by the authors in [4]. To measure the weight of the sample, a laboratory scale (KERN ABT 5NM (KERN&Sohn GmbH, Balingen, Germany); maximum weight of 100 g; discreteness of 0.000001 g; serial number: WB22G0101; calibration certificate number: B61-389-2023-03/1, 24 March 2023) was used.

## 3. Results and Discussion

### 3.1. Morphology

**Figure 3:**
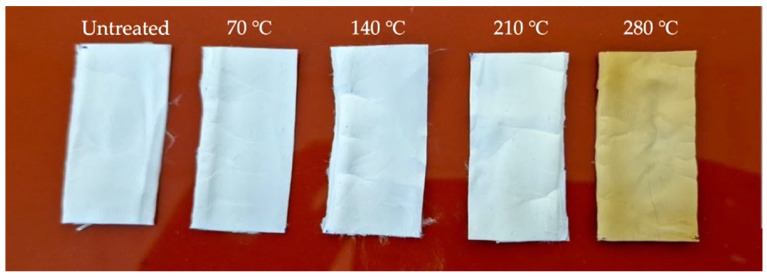
Samples of PAN nanofiber mats after annealing.

Figure 3 illustrates the visual appearance and result of annealing on nanofiber mats. The untreated nanofiber mats and those annealed at 70 °C and 140 °C were both white in color. Nanofiber mats annealed at 210 °C showed a pale yellow color; those annealed at 280 °C appeared golden, which is due to the beginning of the degradation and those annealed at 350 °C appeared to be thoroughly degraded and black. Nanofiber mats annealed at 350 °C were so frail that it was impossible to perform thermal or tensile tests. During annealing, the PAN nanofiber mats changed to light yellow and brown, a phenomenon also observed in our earlier study [3].

**Figure 4:**
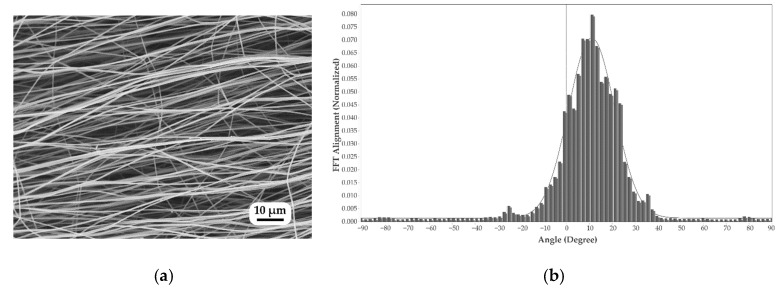
Morphology of nanofibers: (**a**) untreated nanofibers and (**b**) orientation of untreated nanofibers.

Figure 4a shows an SEM image of the untreated nanofiber mat upon which FFT analysis (fast Fourier transform) was performed. Figure 4b shows the results of an investigation into the alignment of the annealed PAN nanofibers. Although the drum revolved at 1800 rpm, the FFT alignment value (normalized) fluctuated from 0.0 to 0.085; most nanofibers were in a single direction. The constant rotational speed stabilized the drawing process and allowed for a consistent diameter range. Anisotropy is a significant factor in determining the mechanical properties of nanofiber mats. When nanofibers are randomly aligned, the mat has less strength than when they are aligned in a single direction. Furthermore, when the rotation speed of the drum collector exceeds 1000 rpm, the resultant nanofibers have a better aligned orientation (compared to rotational speeds less than 1000 rpm) [4,55].

**Figure 5:**
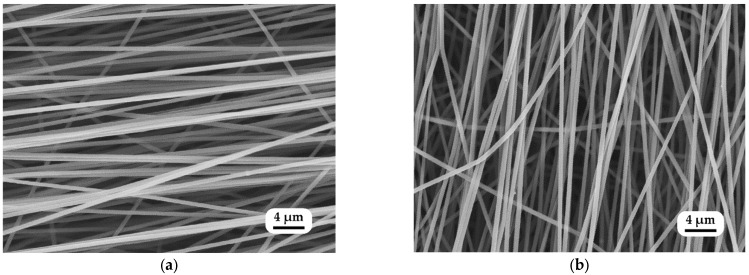
SEM image of nanofiber mats and changes in the diameter of the nanofibers: (**a**) untreated and (**b**) annealed at 140 °C.

Figure 5a,b illustrate SEM images of control samples of PAN nanofibers before and after annealing at +140 °C. The average diameter of an untreated electrospun PAN nanofiber mat was 532 ± 28 nm. Annealing at 70 °C resulted in a slight change in nanofiber diameter, namely, 526 ± 32 nm. It dropped instantly to 434 ± 21 nm when it was annealed at 140 °C, after which it remained stable, and no change was observed until it deteriorated at 350 °C.

**Figure 6:**
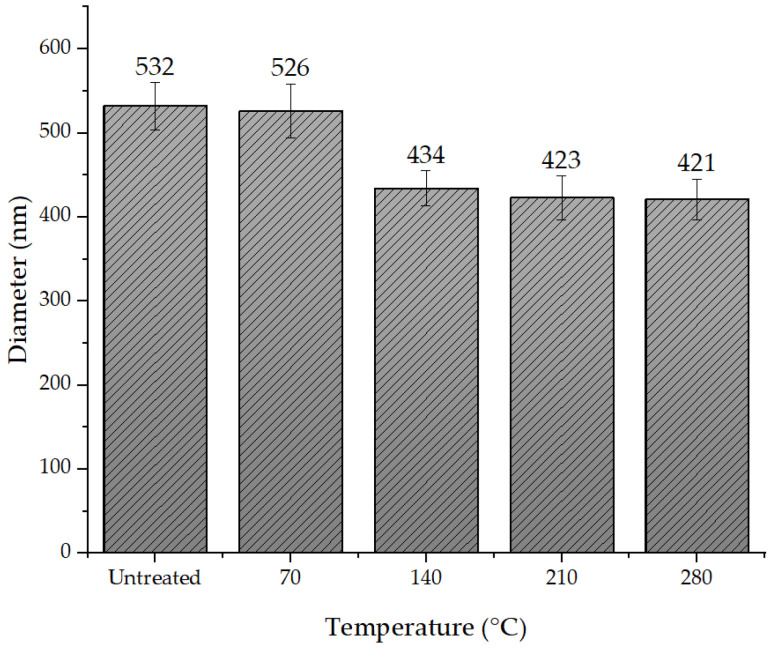
Relationship between the nanofiber diameter and annealing.

The diameters at 210 °C and 280 °C were 423 ± 21 nm and 421 ± 24 nm, respectively. Figure 6 shows the relationship between nanofiber diameter and annealing. Based on the calculated *p*-values, it was concluded that at a significance level of 0.05, there was a significant difference between the diameter values of the nanofiber mat annealed at 70 °C and 140 °C.

The findings of the study revealed that the annealing temperature substantially influences the diameter of electrospun polyacrylonitrile (PAN) nanofiber mats. On average, the diameter of the untreated nanofibers was determined to decrease by around 20% when annealed at 140 °C. The diameter continued to decline as the annealing temperature increased until it stabilized at 210 °C, after which it did not change until it degraded at 350 °C. Similarly, various research groups have found that an annealed polymer nanofiber mat reduces the diameter, and due to the nanofibers and shrinkage, the packing density of the nanofibers also increases [48,56,57].

There are several reasons for the reduction in the diameter of the nanofibers when annealed at different temperatures. First, the PAN nanofibers fabricated via electrospinning require the polymer to be dissolved in a solvent. The solvent may evaporate when heated, causing the fibers to contract as the solvent evaporates [48]. Second, PAN is a thermoplastic polymer that degrades when exposed to high temperatures. When heated, the polymer chains break down, decreasing the fiber diameter due to easier space filling [58]. Third, as the temperature rises, the polymer chains begin to contract, decreasing the fiber diameter, and the polymer chains transition from a solid state to a more flexible, liquid-like phase. Overall, the diameter of heated PAN nanofibers decreases because of shrinkage, molecular rearrangement, and solvent evaporation. The degree to which each component contributes to the decrease in fiber diameter will depend upon the processing circumstances, such as temperature, heating rate, and processing time.

### 3.2. Mechanical Properties

**Figure 7:**
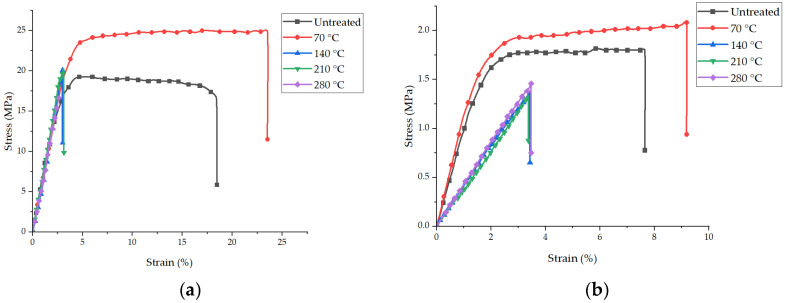
Representative stress–strain graphs of oriented PAN nanofiber mats: (**a**) longitudinal direction; (**b**) transverse direction.

Figure 7a,b shows the stress (σ)–strain (ε) graphs of the specimens of the untreated PAN nanofiber mats and the nanofiber mats after annealing in the longitudinal and transverse directions, respectively, while Table 1 represents the average values. During the tensile test, the slight clamping pressure prevented any failure of the specimens in the grips. The stress–strain graphs reveal that the strength of the annealed PAN nanofiber mat at 70 °C was greater than that of the untreated PAN nanofiber mat. The tensile strength of an untreated PAN nanofiber mat was 19.1 ± 3 MPa, while Young’s modulus was 610 ± 30 MPa. Instantaneous peaks in tensile strength and Young’s modulus of an annealed PAN nanofiber mat at 70 °C were 154 ± 5 MPa and 25.2 ± 2 MPa, respectively. Elongation at break increased from 18.2 ± 2 to 23.6 ± 2%. All PAN nanofiber mats annealed above glass transition temperatures of 90 °C exhibited constant decreases in tensile strength, Young’s modulus, and elongation at break. The PAN nanofiber mat annealed above the glass transition temperature of 140 °C showed a tensile strength of 19.5 ± 1 MPa, Young’s modulus of 598 ± 26 MPa, and an elongation at break of 3.1 ± 0.2%. At 210 °C and 280 °C, the tensile strength values of the annealed PAN nanofiber mats were 19.7 ± 1 MPa and 16.8 ± 1 MPa, and Young’s modulus values were 595 ± 24 MPa and 596 ± 23 MPa, respectively. The changes in the mechanical properties of the nanofibers when untreated and annealed at 70 °C were statistically significantly different. The thickness of the nanofiber mat annealed at 140 °C decreased significantly from 154 ± 5 µm to 121 ± 5 µm. A similar trend was also observed in the transverse direction: tensile strength at 70 °C increased by 23%, and elongation at break increased by 21%, whereas the elastic modulus increased by 18%.

For nanofibers collected in a rotating drum collector at speeds higher than 1000 rpm, the increase in tensile strength was attributable to the improved orientation [55,59,60]. Young’s modulus and tensile strength of nanofibers increased as their diameter decreased significantly below 700 nm. Studies of nanofibers have shown that mechanical properties increase exponentially as the fiber diameter approaches a critical value (below 700 nm). The interwoven nanofiber structure of the nanofiber has fewer imperfections and a more uniform structure, resulting in excellent durability [3,61,62,63]. According to our findings, the average diameter of PAN electrospun nanofibers was 532 ± 28 nm, and their tensile strength and Young’s modulus were 19.1 ± 3 MPa and 610 ± 30 MPa, respectively (Table 1), which corresponds to the authors’ previous results with oriented PAN nanofibers [31].

Annealing is the process of heating and cooling materials to alter their physical and mechanical characteristics [34]; it affects polymers by altering their crystallinity [64]. The annealing process can enhance the modulus and tensile strength of undrawn materials. Moreover, changes in the mechanical characteristics of annealed polymers are sometimes inversely proportional to the annealing temperature. Noticeable changes could be observed near the glass transition temperature [65]. The improvement in mechanical performance may be a consequence of the improved crystallinity. In our results, we found a drop in nanofiber diameter of about 20% at 140 °C, an increase in tensile strength (from 19.1 ± 3 MPa to 25.2 ± 2 MPa), and a rise in Young’s modulus (from 610 ± 30 MPa to 650 ± 21 MPa) at 70 °C. Similarly, other research groups [31,43,44,45] have found that the annealing of nanofiber mats fabricated with various polymers enhanced their mechanical characteristics.

Since heating can further align the polymer chains in the nanofibers, leading to a higher chain orientation, tensile strength can be improved [49,57]. The research findings indicated that subjecting PAN nanofibers to annealing temperatures exceeding the glass transition temperature (Tg), specifically at temperatures of 140 °C and above, led to a notable reduction in their mechanical properties. It is postulated that the observed decline in mechanical properties could be ascribed to the volatilization of the solvent employed in the electrospinning procedure when subjected to temperatures exceeding 100 °C. During the annealing process, the application of high temperatures induces the evaporation of the solvent contained within the nanofiber mat, which, in turn, induces alterations in the structure and morphology of the nanofibers. During the process of solvent evaporation, the nanofibers undergo contraction and rearrangement, leading to a higher packing density and a decrease in intermolecular interactions.

The exposure of PAN nanofibers to oxygen during the annealing process can result in oxidation reactions, which subsequently induce alterations in the chemical structure and properties of the fibers. The process of oxidation can lead to the deterioration of the polymer chains, resulting in a decrease in the overall mechanical integrity of the nanofibers. The introduction of oxygen has the potential to induce a breakdown of polymer chains, thereby weakening the intermolecular forces responsible for the nanofiber mat’s mechanical characteristics. The process of degradation can lead to a reduction in tensile strength, Young’s modulus, and strain.

In summary, heating PAN nanofibers at 70 °C helps to enhance their mechanical characteristics by improving the orientation of the chain and density of the material, resulting in increased tensile strength, elongation, and Young’s modulus. Additionally, annealing above the glass transition temperature or up to the evaporation of the solvent can lead to the degradation of the mechanical characteristics of the nanofiber mat.

### 3.3. Thermal Properties

**Figure 8:**
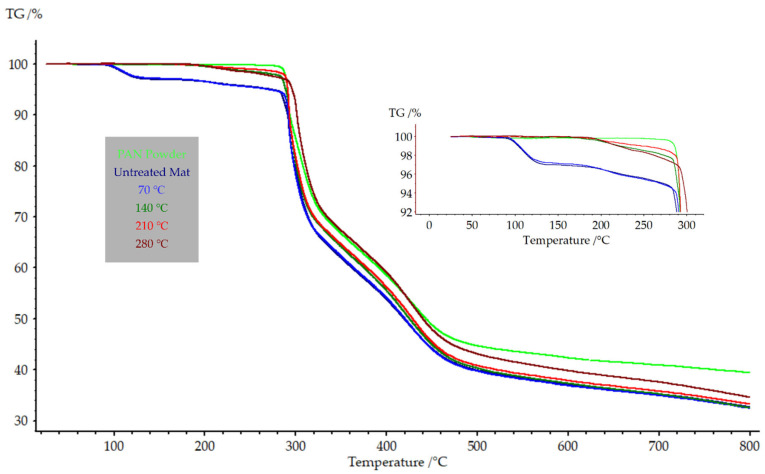
TGA of PAN nanofiber mats annealed at different temperatures.

Figure 8 illustrates the TGA graph of PAN powder, the untreated nanofiber mat, and the annealed nanofiber mats. A distinctive, sharp decline in weight (mass loss up to 30%) was observed in all TGA graphs at approximately 290 °C, indicating the sudden degradation of PAN in the crucible. The PAN powder degraded at 291.6 °C, while the nanofiber mats annealed up to 210 °C showed degradation around the same temperature. The PAN nanofiber mat annealed at 280 °C exhibited degradation at 301.8 °C. Notably, both the untreated nanofiber mat and the nanofiber mats annealed at 70 °C displayed a common mass loss at 100 °C, which could be attributed to the evaporation of the solvent. The solvent evaporation process was initiated at 100 °C and completed at 120 °C. Following the complete evaporation of the solvent, a constant mass was observed until the nanofiber mat underwent complete degradation.

**Figure 9:**
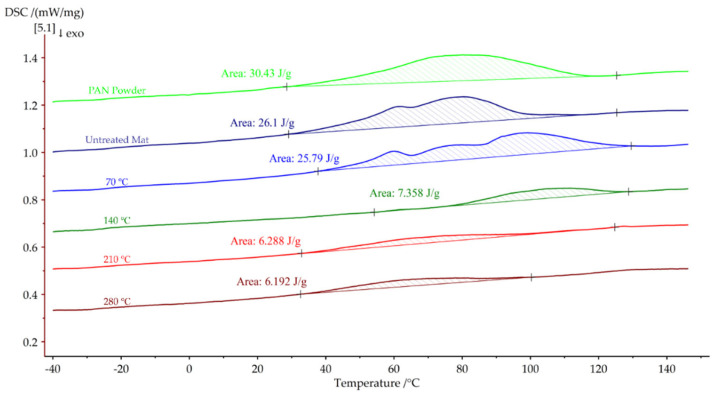
First DSC heating cycles of PAN nanofiber mats annealed at different temperatures.

Differential scanning calorimetry (DSC) was conducted to investigate the glass transition temperature of PAN powder and nanofiber mats. Figure 9 illustrates the results obtained during the first DSC heating cycle. It was observed that evaporation occurred during the first heating cycle, indicating that the observed heat absorption did not correspond to the glass transition temperature. The PAN powder exhibited a heat absorption of 30.43 J/g during the first heating cycle. Similarly, significant heat absorption was observed in the untreated nanofiber mat and the nanofiber mat annealed at 70 °C, with values of 26.1 J/g and 25.79 J/g, respectively. In contrast, nanofiber mats annealed above 120 °C, where solvent evaporation had already occurred, exhibited lower heat absorption during the first heating. Specifically, the nanofiber mats annealed at 140 °C, 210 °C, and 280 °C displayed heat absorption of 7.35 J/g, 6.288 J/g, and 6.19 J/g, respectively.

**Figure 10:**
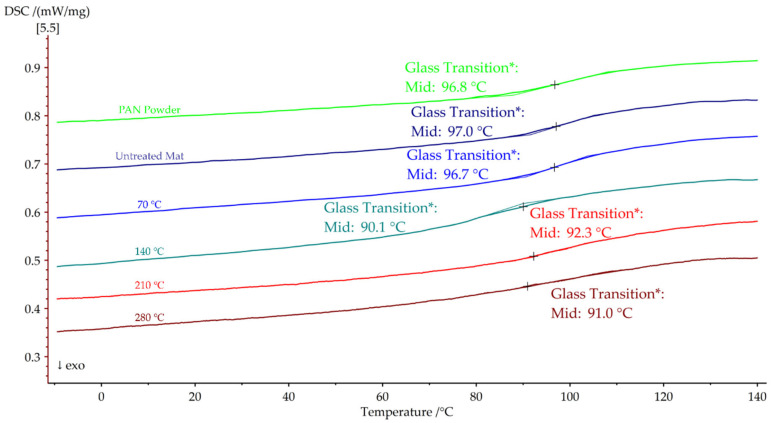
Second DSC heating cycles of PAN nanofiber mats annealed at different temperatures.

The second heating cycle of PAN in the DSC is depicted in Figure 10. The glass transition temperature of PAN powder was initially observed at 96.8 °C. Similarly, the untreated nanofiber mat and the nanofiber mat annealed at 70 °C exhibited glass transition temperatures within a similar range, at 97.0 °C and 96.7 °C, respectively. Notably, nanofiber mats that were annealed above the glass transition temperature displayed a significant decrease in the glass transition temperature. Specifically, the nanofiber mats annealed at 140 °C, 210 °C, and 280 °C exhibited glass transition temperatures of 90.1 °C, 92.3 °C, and 91.0 °C, respectively.

Table 2 illustrates the relationship between the annealing temperature and the glass transition and degradation temperatures. It reveals that as the annealing temperature increases, there is a decrease of approximately 3.5% in the glass transition temperature and an increase of approximately 6% in the degradation temperature compared to the untreated PAN nanofiber mats.

Annealing of the PAN nanofiber mats at various temperatures decreased their Tg values: at annealing temperatures from 70 °C to 280 °C, the Tg decreased from 96.7 °C to 91.0 °C. This decrease in Tg may be attributable to a reduction in fiber diameter caused by the annealing procedure. Slight degradation of PAN occurred due to the cross-linking reaction [66], which was also likely due to thermal treatments being performed at temperatures high enough to induce significant chemical modifications in PAN macromolecules. At the glass transition temperature, the material changed from flexible and rubbery to stiff. The change to a stiff material also explains why the annealed nanofiber mat’s elongation was reduced.

In summary, TGA demonstrated a distinct reduction in weight at approximately 290 °C, suggesting the degradation of PAN. The degradation temperature was found to be influenced by the annealing temperature, whereby higher annealing temperatures were observed to result in elevated degradation temperatures. The results obtained from the DSC analysis conducted during the initial heating cycle exhibited heat absorption that did not correspond to the glass transition temperature. However, the glass transition temperature was observed for both PAN powder and nanofiber mats during the second heating cycle. The glass transition temperature appeared to be influenced by the annealing temperature, whereby higher annealing temperatures were found to correspond to lower glass transition temperatures.

## 4. Conclusions

The research findings indicate that annealing has a significant impact on the mechanical and thermal characteristics of PAN-oriented nanofiber mats produced through electrospinning. The annealing process led to changes in the diameter, tensile strength, Young’s modulus, elongation at break, glass transition temperature, and degradation temperature of the nanofiber mats.

Annealing at temperatures ranging from 70 °C to 280 °C resulted in a decrease in nanofiber diameter of approximately 20%. The decrease in diameter was attributed to thermal degradation, shrinkage, and solvent evaporation. The diameter remained stable after annealing at 140 °C until it degraded at 350 °C.

The tensile strength and Young’s modulus of the annealed PAN nanofiber mats varied depending on the annealing temperature. Annealing at 70 °C increased the tensile strength by 32% and Young’s modulus by 18%. However, annealing above the glass transition temperature (140 °C and above) resulted in a decrease in tensile strength and Young’s modulus. This decrease was attributed to solvent evaporation, contraction, rearrangement, and degradation of the nanofibers. The annealed nanofiber mats also exhibited changes in elongation at break at various temperatures in longitudinal and transverse directions.

The glass transition temperature of the nanofiber mats decreased as the annealing temperature increased, with a decrease of approximately 3.5% at 280 °C, compared to the untreated mats. The degradation temperature showed a slight increase of approximately 6% at 280 °C. The changes in these thermal characteristics were attributed to solvent evaporation, molecular rearrangement, and oxidation reactions.

Overall, the mechanical characteristics of annealing PAN nanofiber mats can be improved at lower temperatures; however, annealing above the glass transition temperature or up to the evaporation of the solvent can lead to degradation of the nanofiber mats. The findings of this research contribute to a better understanding of the effects of annealing on PAN nanofiber mats and their potential applications in various filtration systems.

## Figures and Tables

**Table 1:** Average mechanical properties of oriented PAN nanofiber mats in longitudinal and transverse directions.

	Annealed PAN Nanofiber Mats	Thickness, t (µm)	Tensile Strength σ at Break (MPa)	Young’s Modulus, E (MPa)	Elongation at Break, ε at Break (%)
Longitudinal	Untreated	156 ± 7	19.1 ± 3	610 ± 30	18.2 ± 2
	70 °C	154 ± 5	25.2 ± 2	650 ± 21	23.6 ± 2
	140 °C	121 ± 5	19.5 ± 1	598 ± 26	3.1 ± 0.3
	210 °C	119 ± 4	19.7 ± 1	595 ± 24	3.1 ± 0.2
	280 °C	114 ± 4	16.8 ± 1	596 ± 23	3.0 ± 0.1
Transverse	Untreated	153 ± 9	1.7 ± 0.1	97 ± 6	7.6 ± 1
	70 °C	154 ± 6	2.1 ± 0.1	115 ± 8	9.2 ± 1
	140 °C	125 ± 5	1.7 ± 0.1	44 ± 3	3.3 ± 0.2
	210 °C	117 ± 4	1.3 ± 0.1	42 ± 3	3.3 ± 0.2
	280 °C	113 ± 5	1.4 ± 0.1	45 ± 2	3.4 ± 0.1

**Table 2:** Thermal properties of the nanofiber mat annealed at different temperatures.

Properties	Untreated		Annealed			
	PAN Powder	Nanofiber Mat	70 °C	140 °C	210 °C	280 °C
TGA (°C)	291.6	292.2	292.6	292.7	292.8	301.8
Heat absorbed during first DSC heating cycle (J/g)	30.43	26.1	25.79	7.358	6.288	6.192
Tg during second DSC heating cycle (°C)	96.8	97.0	96.7	90.1	92.3	91.0

## Data Availability

The original contributions presented in the study are included in the article, and further inquiries can be directed to the corresponding author.
